# Supplementary material for: Lysophosphatidic Acid Receptors LPAR5 and LPAR2 Inversely Control Hydroxychloroquine-Evoked Itch and Scratching in Mice
Source: Int J Mol Sci. 2024 Jul 26;25(15):8177. doi: 10.3390/ijms25158177 (PMC11312285; doi:10.3390/ijms25158177)
Supplement: Supplementary file 1 [file ijms-25-08177-s001.zip › ijms-3079634-supplementary.pdf]

Supplementary tables

Supplementary Table S1

Genotyping primer

Name	Sequence	Allele	Length
**LacZ_2_small_F**	ATCACGACGCGCTGTATC L	LacZ	108
**LacZ_2_small_R**	ACATCGGGCAAATAATATCG	LacZ	108
**CRE Forward**	GAAAGCAGCCATGTCCAATTTACTGACCGTAC	Cre	
**CRE Reverse**	GCGCGCCTGAAGATATAGAAGA	Cre	

Supplementary Table S2

RT-PCR primer

Primer name	Sequence	Source
**MRGPRA3 fwd**	5 ’CTCAAGTTTACCCTACCCAAAGG 3‘	Harvard PrimerBank
**MRGPRA3 rev**	5‘ CCGCAGAAATAACCATCCAGAA 3‘	Harvard PrimerBank
**MRGPRC11 fwd**	5‘ TCTCATCCCACGACACAGAAT 3‘	Harvard PrimerBank
**MRGPRC11 rev**	5‘ AGCCAGAGTACAATGGTGTTTC 3‘	Harvard PrimerBank
**LPAR2 fwd**	5‘ GGCCGTGTGGTCACACTC 3‘	(Contos et al. 2002)
**LPAR2 rev**	5‘ CCCAGAATGATGACAACCGTCTT 3‘	(Contos et al. 2002)
**LPAR5 fwd**	5‘ CAAGAAGGTCTCCACTGCTGA 3‘	
**LPAR5 rev**	5‘ GTGGTAGCCTGGTGGCAATA 3‘	

Supplementary Table S3

Primary antibodies and fluorophore conjugates

Antibody	Manufacturer	Product #	Dilution	Host	Type	Use
**Beta Gal**	Abcam	Ab116	1:500	mouse	mab	IFL
**Beta Gal**	Abcam	Ab9361	1:200	chicken	pab	IFL
**GAD65 and GAD67**	Abcam	Ab49832	1:200	rabbit	pab	IFL
**Homer**	SCBT	Sc-15321	1:200	rabbit	pab	IFL
**IB4 Alexa 488**	Invitrogen	I 21411	1:200			IF
**IB4 Alexa 594**	Invitrogen	I21413	1:200			IF
**MrgprX1**	Abcam	Ab77519	1:200	Rabbit	pab	IFL
**NeuN**	Abcam	Ab177487	1:500	rabbit	mab	IFL
**Peripherin**	Chemicon	Ab1530	1:50	Rabbit	pab	IFL
**Tuj-1**	Promega	G7121	1:500	Mouse	mab	IFL

IFL, Immunofluorescence; FL, fluorescence; mab, monoclonal antibody; pab, polyclonal antibody

Supplementary figures

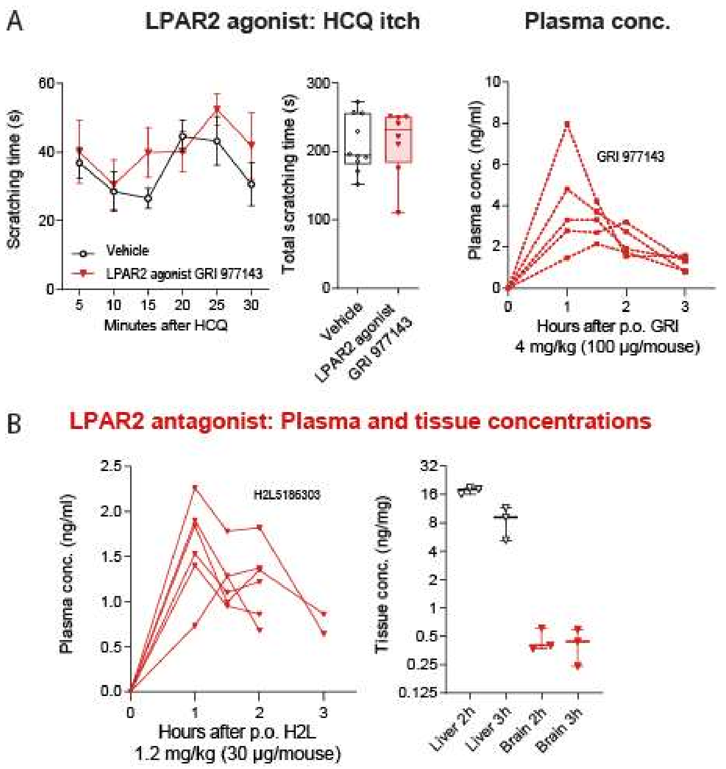

Supplementary Figure S1

Itch behavior and drug concentrations in plasma and tissue

A: Scratching behavior in mice treated with the LPAR2 agonist GRI 977143 (100 µg/mouse; ≈ 4 µg/g) and plasma concentrations after oral administration of the drug. Scratching behavior was evoked by intradermal injection hydroxychloroquine (HCQ) (n = 8 per group). Plasma concentration time courses were measured by HPLC-MS-MS analysis of n = 5 per group.

B: Plasma and tissue concentrations of the LPAR2 antagonist H2L5186303 upon oral administration of 30 µg/mouse (≈ 1.2 µg/g) in n = 6 mice per group. The group was split to assess liver and brain concentrations at 2 and 3 hours.

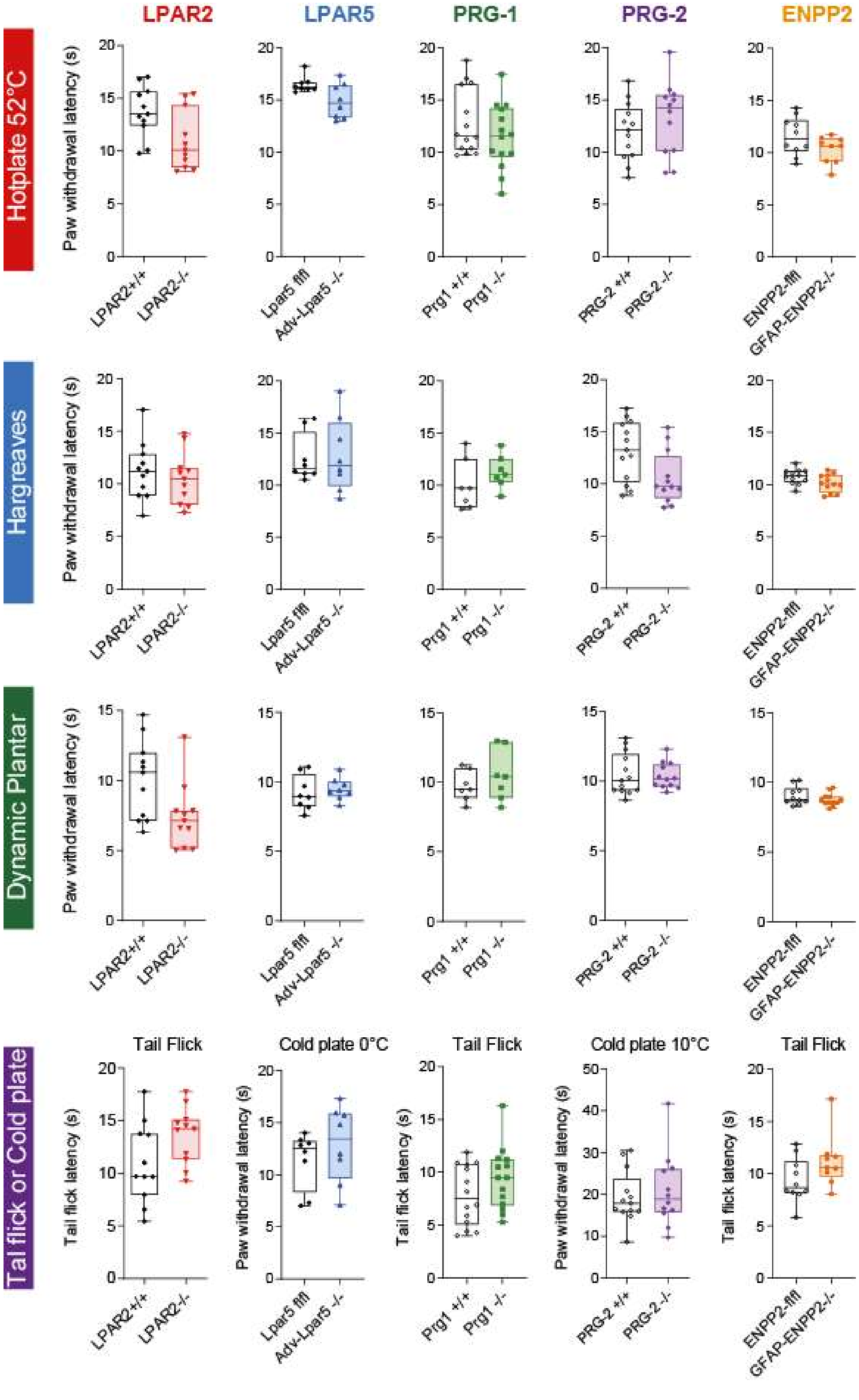

LPAR2
LPAR5
PRG-1
PRG-2
ENPP2
Hotplate 52°C
Hargreaves
Dynamic Plantar
Tal flick or Cold plate
Paw withdrawal latency (s)
Tail flick latency (s)
Tail Flick
Cold plate 0°C
Tail Flick
Cold plate 10°C
Tail Flick
LPAR2+/+
LPAR2-/-
Lpar5 fl/fl
Adv-Lpar5 -/-
Prg1 +/+
Prg1 -/-
PRG-2 +/+
PRG-2 -/-
ENPP2-fl/fl
GFAP-ENPP2-/-

Supplementary Figure S2

Basic nociceptive behavior upon thermal or mechanical stimulation

Paw withdrawal latencies were measured upon heat stimulation on a 52°C Hotplate, upon mechanical stimulation with a dynamic von Frey aesthesiometer, upon thermal stimulation with the Hargreaves test, upon heat stimulation of the tail (tail flick) or cold stimulation on a cold plate. The genotypes were LPAR2$^{-/-}$ versus wildtype, Advillin-LPAR5$^{-/-}$ versus floxed controls (LPAR5-flfl), Prg1/LPPR4$^{-/-}$ versus wildtype, Prg2/LPPR5$^{-/-}$ versus wildtype and GFAP-ENPP2$^{-/-}$ versus ENPP2-flfl controls (ENPP2 = autotaxin). The boxes show the interquartile range, the line is the median, whiskers show minimum to maximum and the scatters represent individual mice. Unpaired 2-tailed Student's t-tests did not show significant differences between the respective genotypes.
